# Usage and health perception of cannabidiol-containing products among the population in Germany: a descriptive study conducted in 2020 and 2021

**DOI:** 10.1186/s12889-023-17142-0

**Published:** 2023-11-23

**Authors:** Johanna Geppert, Julika Lietzow, Stefanie Hessel-Pras, Fabian Kirsch, Bernd Schäfer, Benjamin Sachse

**Affiliations:** German Federal Institute for Risk Assessment (BfR), Max-Dohrn-Str. 8–10, Berlin, 10589 Germany

**Keywords:** Cannabidiol, CBD, Consumption, Risk perception, Online survey, Germany

## Abstract

**Background:**

Cannabidiol (CBD), a non-intoxicating substance of *Cannabis sativa L*., is gaining consumer attention. Yet, legal regulations in the EU are complex and questions of potential health risks remain partly unanswered. In Germany, little is known about people who use CBD products. The aim of this cross-sectional study was to gain insight into the user group of CBD, reasons for consumption and risk perception towards CBD-containing products.

**Methods:**

The study consisted of two parts: In the first part of the study, the prevalence of CBD awareness and usage in Germany was estimated using a telephone survey and a population-representative sample of *n* = 1,011 respondents. Based on these results, *n* = 2,000 participants being aware of CBD were surveyed with an online questionnaire in the second part of the study to examine usage and perception of CBD in users and non-users.

**Results:**

When the study was conducted at the end of 2020 and beginning of 2021, 40.2% of the German participants had already heard of products containing CBD, and 11.4% had actually used them. 42.1% of the users consumed such products regularly, at least once a week, primarily orally via oils or tinctures, and purchased them mainly online. Besides curiosity – addressed especially in young adults – anticipated health benefits including pain and stress relief were main reasons for use. More than half of the study participants perceived the health benefits of CBD use as high or very high. In contrast, the health risks were rated as low or very low by most respondents. Assumptions about official testing for safety as well as physical effects of CBD-containing products varied between users and non-users.

**Conclusion:**

About one in nine people in Germany uses CBD-containing products. Given reasons for consumption and perception of potential health risks and benefits suggest that people are insufficiently informed about CBD-containing products. The results of the study indicate that risk communication is needed to raise awareness for the topic and to inform (potential) users.

**Supplementary Information:**

The online version contains supplementary material available at 10.1186/s12889-023-17142-0.

## Introduction

In recent years, cannabidiol (CBD) is gaining growing consumer attention [1]. The substance belongs to one of at least 130 naturally occurring phytocannabinoids that have been identified in *Cannabis sativa *L. [2]. In industrial hemp, CBD is the main cannabinoid [3]. In contrast to tetrahydrocannabinol (THC), CBD is not intoxicating [4] and – according to a decision of the Court of Justice of the European Union – should therefore not be considered a narcotic [5]. Notwithstanding, CBD interacts with several molecular targets in the organism, potentially resulting in beneficial but also adverse health effects [6].

In consequence, products containing high CBD doses clearly exerting a pharmacological activity or being intended for the treatment of diseases are considered to be medicinal products in the European Union (EU). They require authorization according to EU regulations on pharmaceuticals [7]. The rationale behind this is to protect consumers from potential health risks that may arise from the products themselves or use of non-approved products with unproven effects instead of effective medicines.

Only low-dose CBD products without a pharmacological activity and without the intention to treat diseases may potentially considered to be foods. Such CBD-containing foods, however, are generally classified as novel in the EU [8]. They require a safety assessment carried out by the European Food Safety Authority (EFSA), followed by authorization by the European Commission before they can be marketed in the EU [9]. Numerous novel food applications for CBD and CBD extracts have been submitted during the last years and are partly under risk assessment [10]. EFSA recently identified some potential hazards but was not able to fully assess the actual risks due to several data gaps. Consequently, it was concluded that “*the safety of CBD as a novel food cannot currently be established”* [11]. Therefore, no foods containing CBD have yet been authorized and the marketing of such products as foods, including supplements, is currently considered illegal. Despite these rules, consumers can buy an increasing number of illegally marketed CBD-containing products, also in the form of food supplements, that are available mostly online or in retail stores. In some cases, such products are mislabeled, for example as “aroma oils” or “cosmetics” to circumvent legal rules [12].

As described, the regulatory context for CBD is complex and it might be put into question whether consumers are aware of both the legal aspects as well as the potential health risks. Therefore, the aim of the current study was to gain insight into the user group of CBD in Germany, to investigate reasons for consumption, and to gain information about the risk perception of the population and its knowledge about CBD-containing products in order to target risk communication and increase awareness for the substance.

## Methods

### Study design and participants

The study was conducted in two parts: In study part I the prevalence for CBD awareness and usage was determined while study part II examined the usage and perception of CBD for users vs. non-users in detail. The two studies consisted of independent samples. Data collection was conducted by German market research institutes. All respondents expressed their consent to participate in the surveys.

Study part I was carried out in Germany via telephone survey from November 25 to November 26, 2020 by trained market research assistants. The survey was conducted with a sample of *n* = 1,011 respondents aged 14 years and older who were randomly selected via a random digit dialing procedure including mobile and landline telephone numbers. For mobile phone numbers, the person answering the call was selected for the interview. For landline telephone numbers, the Kish selection method [13] was used to choose the respondent within a household. To achieve population representativeness, data were statistically weighted using an iterative process and a 2-step-procedure: [14] In a first step, weighting took into account the number of mobile and landline telephone numbers respondents could be reached by (design weighting). In a second step, data were weighted based on the German microcensus regarding gender, education, age, employment, size of household and city, West-/East-Germany and German federal state (adjustment weighting). Within the questionnaire, participants were asked whether they had already heard of products containing CBD and, if so, whether they had consumed or used such products (Suppl. A, Questionnaire Q1).

Study part II was carried out in Germany via an online panel survey from March 11 to March 23, 2021, using a quota sample. Quotas for gender, age, and education were determined based on the results of study part I. Respondents were randomly selected from the panel until the desired number of participants meeting the quota targets were obtained. An online questionnaire was developed to determine CBD-containing product consumption patterns, frequency and reasons for use, the perception of health risks and benefits, and knowledge about CBD-containing products. Respondents received remuneration in accordance with the usual incentive structure of a panel. The sample consisted of *n* = 2,000 respondents with an age of 16 years and older that indicated that they had already heard of products containing CBD.

### Online questionnaire

The online questionnaire of study part II included questions on sociodemographic measures such as gender, age, and education, as well as use, product groups, its purchase, and frequency of use. Additional questions addressed the intention of future use and the perception of health risks and benefits. Furthermore, open questions asked participants about reasons for use and assumed health risks and benefits. Responses to these open-ended questions were coded using code frames that were developed within an inductive process. Multiple codes were used for responses that consisted of more than one aspect. The coding was cross-validated by two researchers.

All questions included “don’t know” and/or “no answer” options. For more details see the questionnaire (Suppl. A).

### Statistical analysis

Data were processed and analyzed using SPSS (Version 26). Descriptive statistics including the calculation of means (*M*) and standard deviations (*SD*) were used to describe socio-demographics as well as product and usage characteristics. In addition to descriptive statistics, differences were analyzed for sociodemographic groups (gender, age, education) and user status (user vs. non-user). For these analyses, we grouped respondents into three age categories (up to 29 years, 30 to 59 years, 60 years and older) as well as three educational categories (low: without graduation / student / lower secondary school; medium: secondary school without high school diploma; high: high school / university degree). Analyses for Study part I are based on a weighted sample (see “Study design and participants”). Therefore, we applied Rao-Scott adjustment [15] for complex survey data for all tests of significance within study part I. Effect sizes were calculated using common thresholds for interpretation (Suppl. B, Table S1).

## Results

### Awareness and usage of products containing CBD

**Fig. 1:**
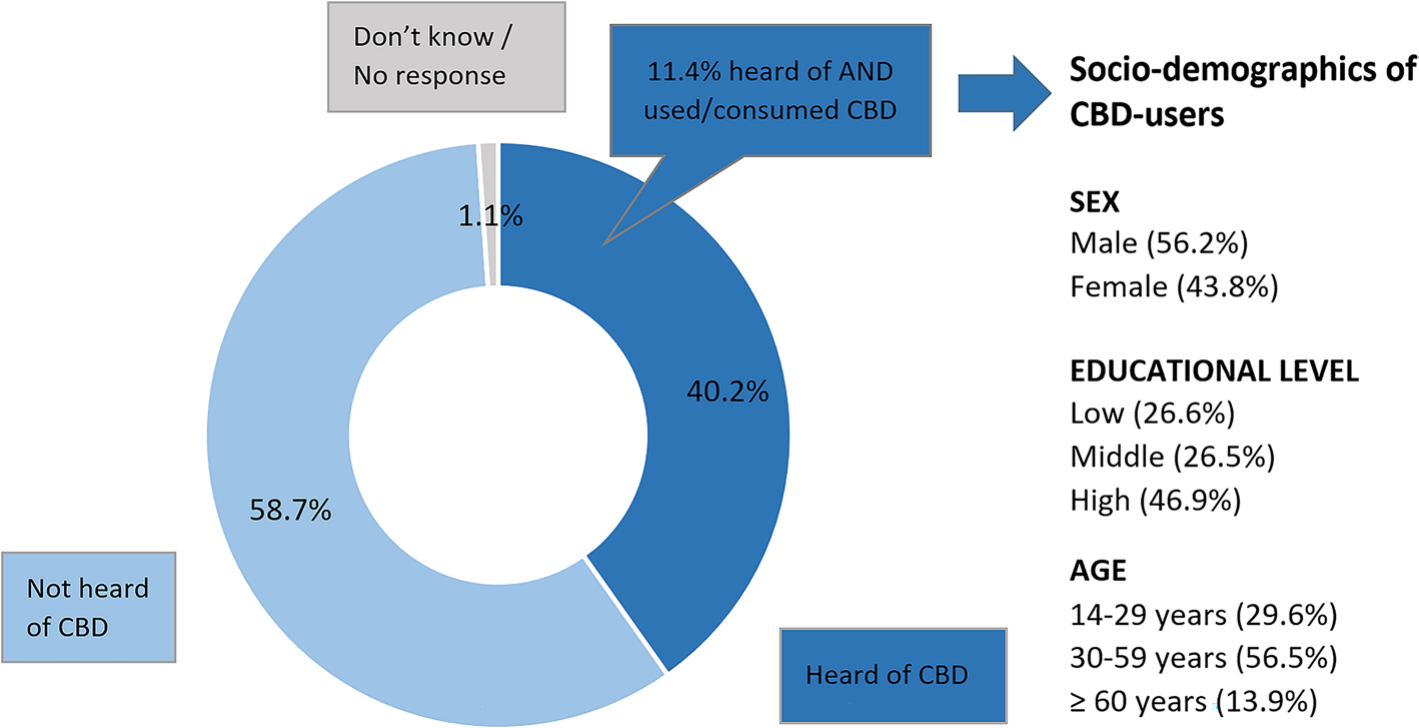
Study part I: Percentage of participants answering “yes”, “no” or “don’t know / no response” to the question “Have you already heard of any products that contain CBD?”, Percentage of participants subsequently answering “yes” to the question “Have you already consumed or used any products that contain CBD?”; Details in Suppl. B, Table S2. Base: All respondent (*n* = 1,011) and respondents who have already consumed/had used CBD (*n* = 116)

Study part I revealed that 40.2% of the participants between 14 and 95 years (*M* = 49.4 years, *SD* = 19.8) have already heard of CBD-containing products. Of them, 28.5% have already used such products. This corresponds to a proportion of 11.4% of the total number of respondents (see Fig. 1). Further analysis for usage behavior revealed sociodemographic differences, with the youngest age group of under 30 years being significantly more likely to use such products than older age groups (*F*[1.7, 1727.7] = 4.79, *V* = 0.15, *p* = .01). There were no significant differences for gender (*F*[1, 1010] = 0.83, *V* = 0.05, *p* = .36) and education (*F*[2, 2013] = 2.30, *V* = 0.11, *p* = .10). Further demographic characteristics of the study part I are summarized in Table S2 of the Supplemental material B.

**Fig. 2:**
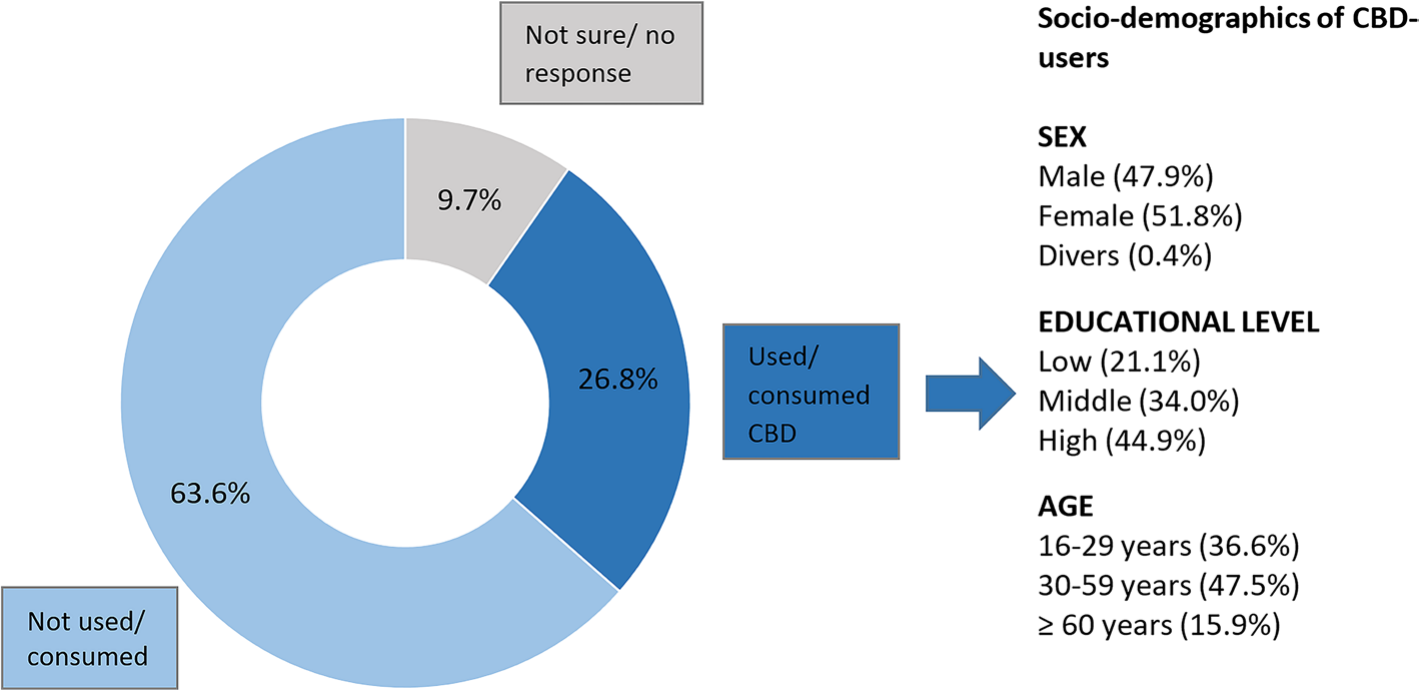
Study part II: Percentage of all participants answering “no”, “not sure / no response”, “yes” to the question “Have you already consumed or used any products that contain CBD?”; Main demographic characteristics of the respondents that already consumed or used any products that contain CBD (details in Suppl. B, Table S3). Base: All respondents (*n* = 2,000) and respondents who have already consumed/had used CBD (*n* = 535)

Based on these findings, 2,000 subjects with prior awareness of CBD-containing products were recruited for study part II. Participants were between 16 and 85 years (*M* = 43.6 years, *SD* = 16.2). 26.8% had prior experience using CBD (Fig. 2 and Suppl. B, Table S3). Those who gave no response or were not sure if they had consumed or used CBD (9.7%) were excluded from analysis comparing users (respondents with prior experience using CBD) vs. non-users (respondents without prior experience using CBD). Non-users (*n* = 1,271) were asked whether they could imagine consuming CBD-containing products in the future, of which 48.3% answered affirmatively (Suppl. B, Table S4).

### Purchase behavior

**Fig. 3:**
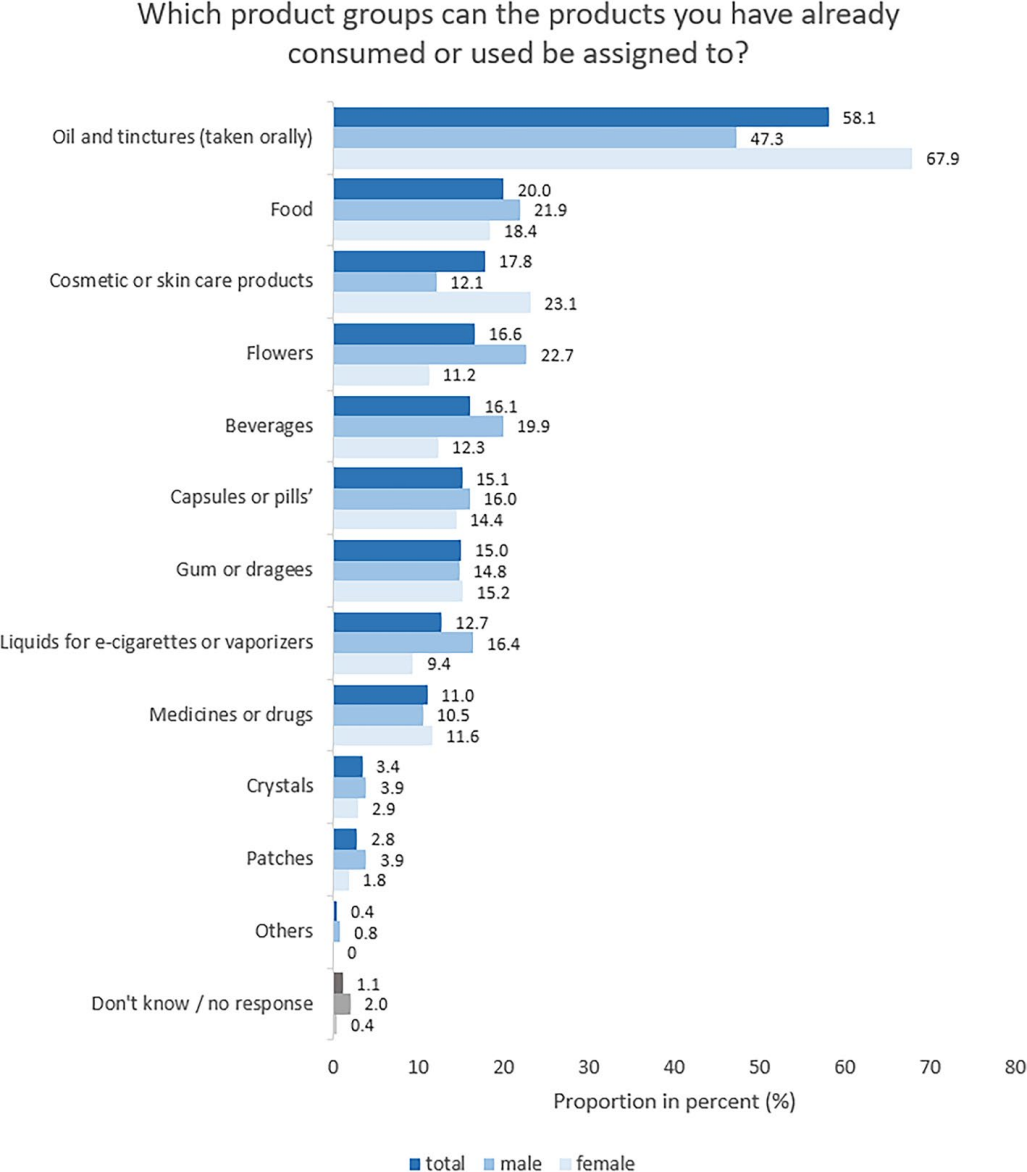
Study part II: Product groups containing CBD used by consumers/users; separated by gender. Base: Respondents who have already consumed/had used CBD (*n* = 535), male (*n* = 256) and female (*n* = 277); multiple answers possible. Respondents indicating “divers” as gender are included in the total sample

 Study part II revealed that of all users (*n* = 535), the majority obtained CBD-containing products via online stores (55.3%). In addition, pharmacies (21.1%), specialized stores for CBD and hemp products (20.0%) and drug stores (19.6%) were also reported as sources of supply (Suppl. B, Table S5). CBD-containing products used included oils and tinctures (58.1%), foods (20.0%), cosmetic and skin care products (17.8%), flowers (16.6%), beverages (16.1%), as well as capsules or pills (15.1%, see Fig. 3). If gender distribution is considered, female users indicated significantly more often than male users that they use CBD-containing oils and tinctures ( Χ² [1] = 23.18, *V* = 0.21, *p* < .001,) as well as skin care products (Χ² [1] = 10.98, *p* < .001), whereas male users reported using CBD-containing flowers ( Χ² [1] = 12.57, *V* = 0.15, *p* < .001), beverages (Χ² [1] = 5.80, *V* = 0.10, *p* = .02), and liquids for e-cigarettes or vaporizers (Χ² [1] = 5.89, *V* = 0.11, *p* = .02) significantly more often than female users. Gender differences were of small effect size.

### Frequency and reasons for use of CBD-containing products

In study part II, when asked about the frequency of use, 42.1% of users indicated that they currently use CBD-containing products at least once a week and 17.6% use CBD-containing products daily. Of those with prior experience with CBD, 19.8% reported that they do not currently use any product.

Considering demographic differences, respondents in higher compared to lower age groups (Χ² [2] = 26.33, *V* = 0.22, *p* < .001) and those with a lower compared to a higher level of education (Χ² [2] = 18.53, *V* = 0.19, *p* < .001) reported more often to consume CBD products at least once a week (Suppl. B, Table S6). Group differences were of medium effect size.

**Fig. 4:**
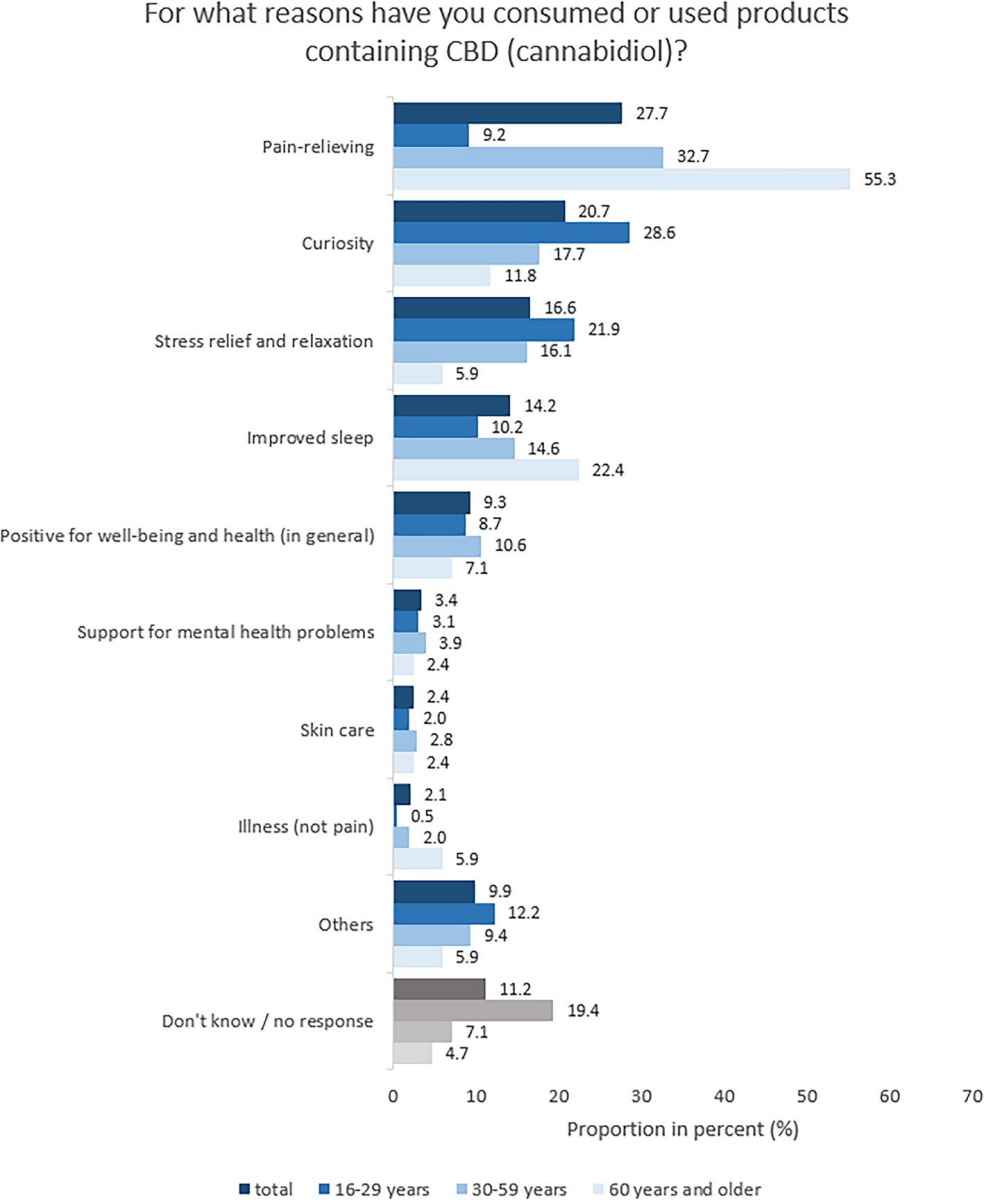
Study part II: Reasons for the consumption or use of CBD-containing products (open question) separated by age groups. Base: Respondents who have already consumed/had used CBD (*n* = 535), 16–29 years (*n* = 196), 30–59 years (*n* = 254) and 60 years and older (*n* = 85)

The main motivation for consuming CBD-containing products stated in an open-ended question is pain relief (27.7%), followed by curiosity (20.7%), stress relief and relaxation (16.6%) as well as improved sleep quality (14.2%, Fig. 4). Reasons for use are differently distributed among the age groups, partly with large effect sizes: Compared to the youngest age group (16–29 years), older age groups significantly more often mentioned pain relief (Χ² [2] = 69.07, *V* = 0.36, *p* < .001) and improved sleep (Χ² [2] = 7.23, *V* = 0.12, *p* = .03). Conversely, the youngest age group mentioned significantly more often curiosity (Χ² [2] = 12.89, *V* = 0.16, *p* = .002) and stress relief/relaxation (Χ² [2] = 11.11, *V* = 0.14, *p* = .004) as reasons for use. Also, the youngest age group was less likely to respond to the question (Χ² [2] = 21.22, *V* = 0.20, *p* < .001).

### Perception of health risks and benefits

**Fig. 5:**
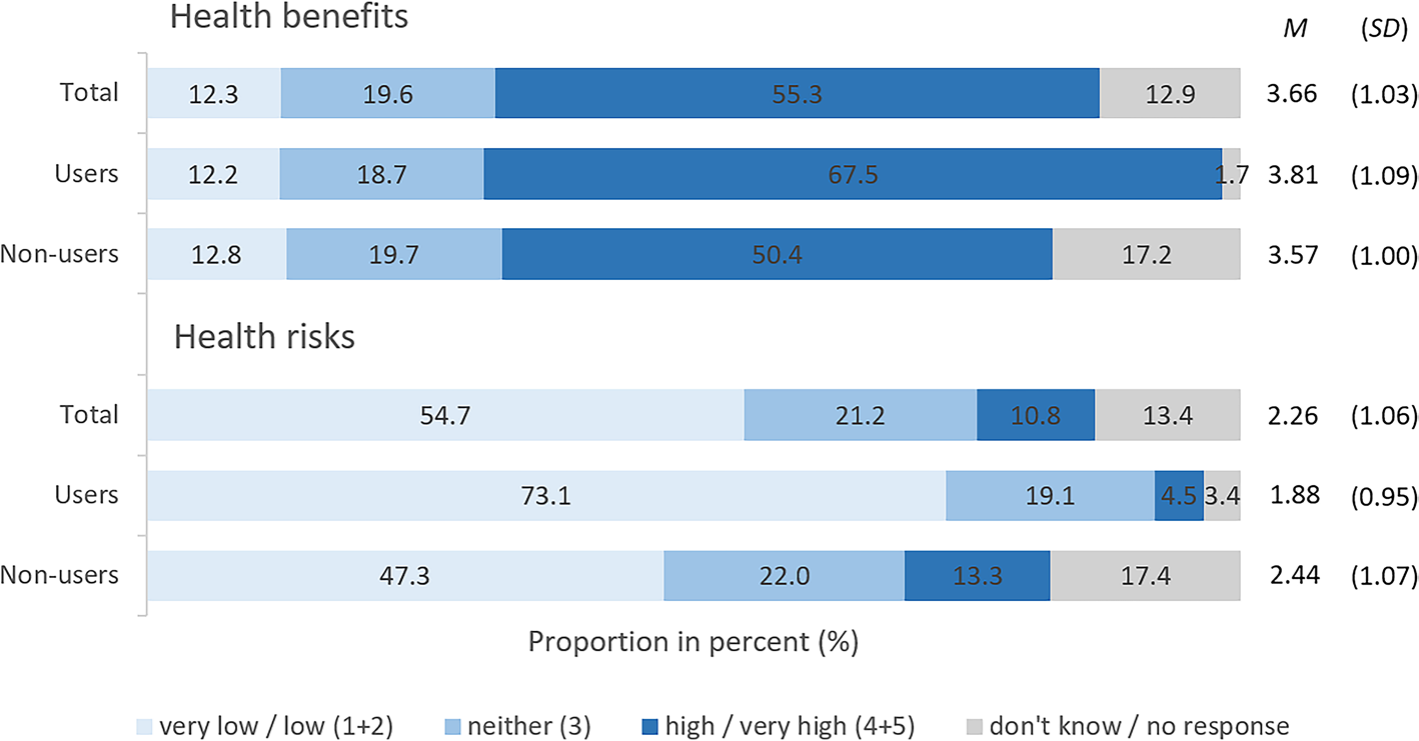
Study part II: Perception of health risks and benefits. Answers to the question “How do you rate the health risks and health benefits of products containing CBD (cannabidiol)?” on a scale from 1 = very low to 5 = very high. Base: All respondents (*n* = 2,000), users (*n* = 535) and non-users (*n* = 1,271)

Across all participants in study part II, health benefits were rated to be much higher than health risks (*t*[1689] = -33.86, *d* = -0.82, *p* < .001; Fig. 5). While 54.7% of all respondents consider the health risks of CBD products to be low or very low, 55.3% perceive the health benefits of CBD-products to be high or very high. Comparing users versus non-users, non-users significantly consider health risks to be greater than users (*t*[1139.4] = -10.51, *d* = -0.54, *p* < .001), whereas users consider health benefits to be somewhat greater than non-users (*t*[1577] = 4.34, *d* = 0.23, *p* < .001). Notably, the percentage of respondents who did not give an answer was higher among non-users than among users for both health benefits (Χ² [1] = 81.99, *V* = 0.21, *p* < .001) and health risks (Χ² [1] = 64.48, *V* = 0.19, *p* < .001), with medium effect sizes. For further details see Suppl. B, Table S7.

**Fig. 6:**
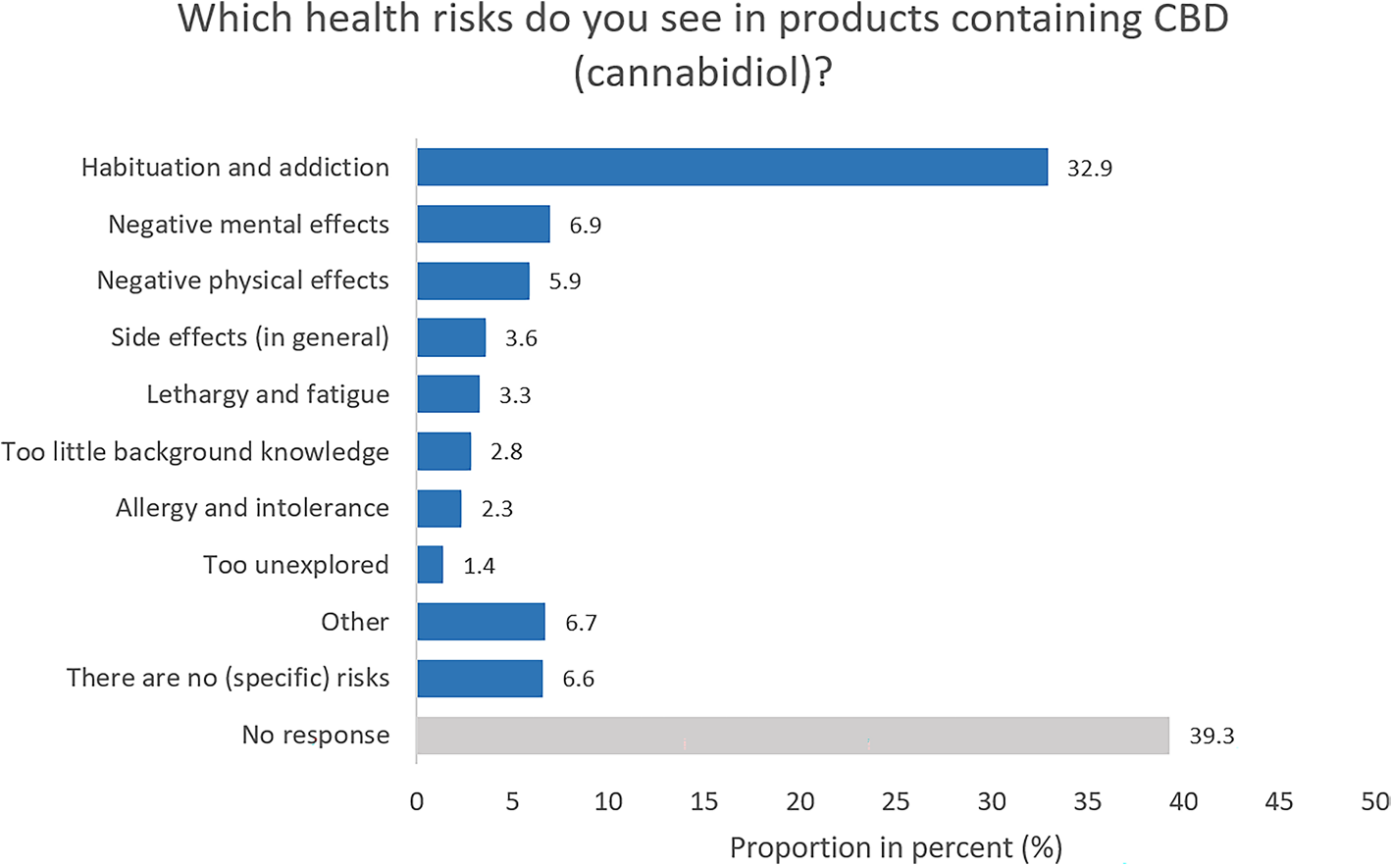
Study part II: Perceived health risks. Base: Respondents who rated health risks of CBD products as at least 3 on a scale of 1 (very low) to 5 (very high) (*n* = 639). Open-ended question

Respondents who rated the health risk of CBD products as at least 3 (on a scale of 1 = very low to 5 = very high) were further asked to name health risks that they see in such products using an open-ended question (*n* = 639; Fig. 6). About one third (32.9%) of the respondents were concerned about habituation and addiction, followed at a great distance by negative mental effects (6.9%) and negative physical effects (5.9%).

**Fig. 7:**
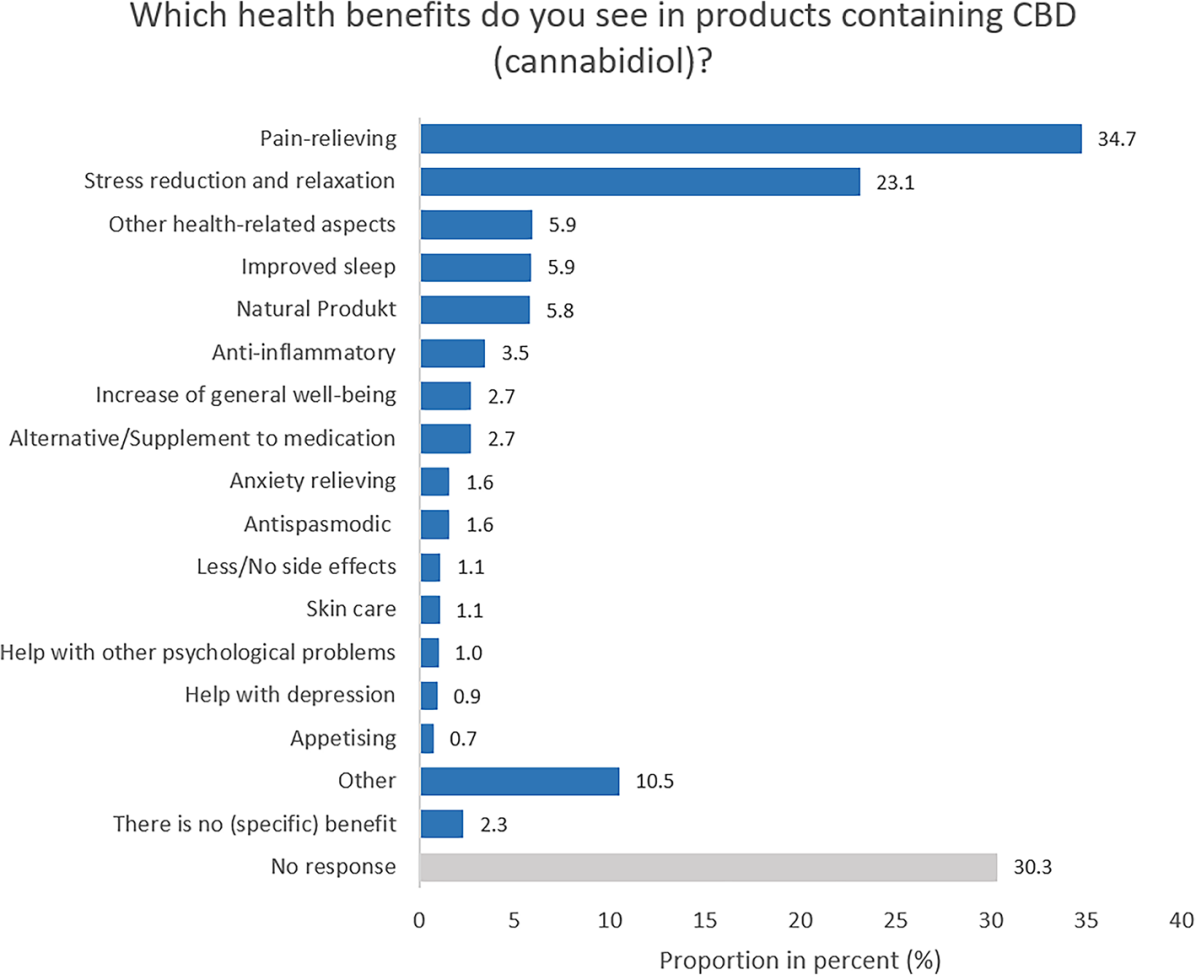
Study part II: Perceived health benefits. Base: Respondents who rated health benefits of CBD products as at least 3 on a scale of 1 (very low) to 5 (very high) (*n* = 1,497). Open-ended question

Respondents who rated the health benefits of CBD products as at least 3 (on a scale of 1 = very low to 5 = very high) were further asked to name health benefits that they see in such products using an open-ended question (*n* = 1,497; Fig. 7). The most commonly cited benefit is the anticipated pain-relieving effect of CBD (34.7%). Just under a quarter of respondents (23.1%) cite stress reduction and relaxation. Further health benefits stated by the respondents were better sleep (5.9%) and the naturalness of CBD (5.8%).

**Fig. 8:**
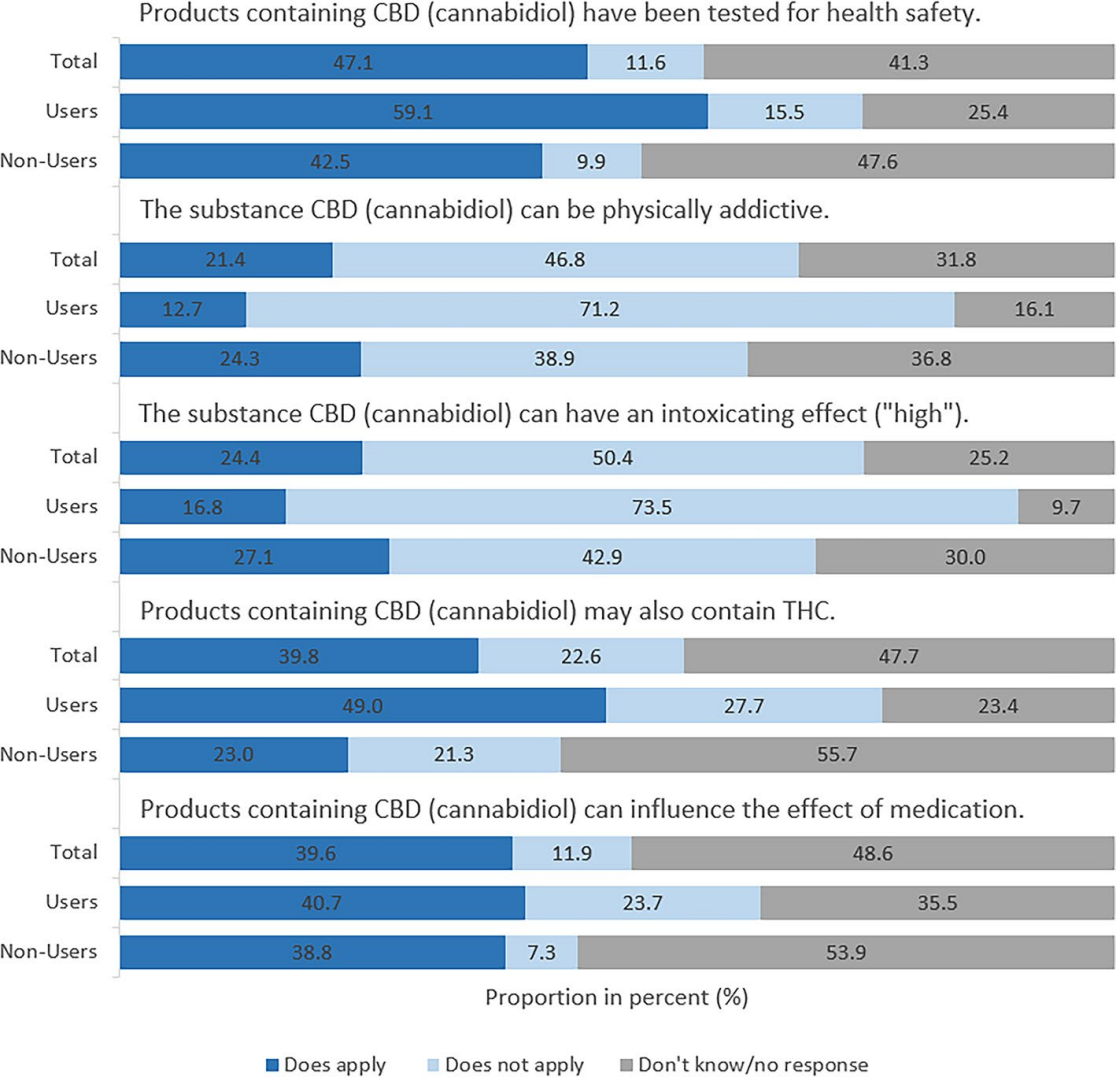
Study part II: Knowledge about CBD. Percentage of respondents answering “In your opinion, does each of the following statements apply or not?” Base: All respondents (*n* = 2,000), users (*n* = 535) and non-users (*n* = 1,271)

In study part II, all participants were asked about general safety aspects of CBD-containing products (Fig. 8). In this context, 47.1% of the respondents assume that CBD-containing products have been tested to ensure their safety. This proportion is somewhat higher amongst users of CBD-containing products than non-users (59.1% vs. 42.5%; Χ² [1] = 41.51, *V* = 0.15, *p* < .001). While 71.2% of users disagree that CBD can be physically addictive, only 38.9% of non-users make that assumption. Considerably more users than non-users assume that CBD cannot provoke a feeling of being “high” (73.5% vs. 42.9%; Χ² [1] = 141.03, *V* = 0.28, *p* < .001) and also think that CBD-containing products may contain THC (49.0% vs. 23.0%; Χ² [1] = 119.67, *V* = 0.26, *p* < .001). 39.6% of all respondents assume that CBD may interact with medicinal products. For all items, proportion for “don’t know / no response” was quite high (between 25.2 and 48.6% across the total sample) and significantly more often indicated by non-users compared to users (Χ²s [1] ≥ 50.93, *V*s ≥ 0.17, *p*s ≤ 0.001).

## Discussion

The results of the study provide insights into general awareness and use of CBD-containing products in the German population. 40.2% of the respondents had already heard of such products. This proportion is slightly lower than observed in other studies. In France, about 69% had already heard of CBD [16], while a recent German study found a proportion of approximately 48% [17]. Looking at the prevalence of users in the different studies, a relatively consistent picture emerges for European countries with about one in ten who already has used CBD-containing products among the general population (Germany: 11.4% according to our study; France: 10.1%.; UK: 10.9%) [16, 18]. In contrast, a substantially lower usage rate of 4.3% was observed for Germany in a recently published study by Alayli et al. [17]. This deviation may be due to a specific focus on recreational use of CBD products in this study whereas our study as well as the other European studies considered a broader product range. In contrast, the proportion of users (just within the past 12 months) was higher in a non-representative study conducted in the US (26.1%) and in Canada (16.2%) [19]. Classification and accessibility varies strongly between these countries [20, 21] which – in part – might explain the varying proportions of users between countries. In addition, due to the generally more liberal approach to cannabis in some parts of the US and Canada, it might also be assumed that consumer acceptance to CBD products is higher in North American Countries than in Europe.

In the present study, the age group under 30 years had the largest proportion of users. This finding is consistent with another study that observed a proportion of approximately 34% users among adults aged 18–24 years in the US [22] pointing out that the use of CBD products is relatively prominent among young adults. These results give valuable insights with regard to relevant target groups for risk communication measures.

More than half of the users interviewed consume CBD orally via oils or tinctures, to a lesser extent via other products like foods or cosmetic and skin care products. Further, almost 13% indicated to use liquids with CBD for e-cigarettes and vaporizers. Interestingly, a study by Alayli et al. [17] showed that awareness and consumption of recreational CBD products is generally associated with e-cigarette use. At the same time, synthetic cannabinoids in e-liquids have been associated with several health risks including neurological symptoms [23]. The use of CBD in this form should therefore receive special attention in risk communication.

Beyond, a relatively large part of the users consume CBD products at least once a week (42.0%), indicating a rather regular than occasional usage pattern. Results from the current study and other studies clearly show that, beside curiosity, anticipated health benefits such as pain-relief, stress reduction, and improvement of sleep are important reasons for consumption [17, 19, 22]. In the present study, main reasons for consumption significantly differed between age groups. While curiosity and stress relief were commonly cited by the youngest age group (16–29 years), pain relief and improved sleep were important motivators for the older respondents, especially those of 60 years and older.

In the present study, health benefits of CBD-containing products were mentioned more often by the participants than potential health risks, especially among users of such products. Similar results were obtained by others [19, 18]. Indeed, scientific data show that CBD interacts with several molecular targets [6]. Therefore, it is frequently assumed that CBD can be used as a therapeutic agent [24]. However, only one medicinal product solely containing CBD as the active ingredient is currently authorized in the US and in the EU, intended for the treatment of certain forms of epilepsy or tuberous sclerosis [20, 25]. Although there are first indications for therapeutic effects also for other disorders, the clinical efficacy of CBD for those indications has not yet been finally proven [26, 27].

Health risks were considered as being low or very low by the majority of participants in the present study, especially among users. Similar observations were made in other studies [16, 19]. However, following the motto “no effects without side effects”, various adverse effects have already been associated with CBD exposure. Results from animal studies and the use as a drug indicate potential hazards like hepatotoxicity, gastrointestinal complaints, neurological symptoms, a negative impact on the endocrine system, reproductive toxicity, as well as potential drug-drug interactions – at least at higher doses [4, 11]. However, due to data gaps health risks cannot be finally evaluated at the time [11]. Interestingly, the most frequently mentioned concern among the respondents, namely addiction, has no relevance from a scientific point of view, as long as products are not contaminated with THC. These results indicate that consumers are insufficiently informed about the scientific uncertainties regarding the effects of CBD. Here, targeted risk communication measures could prevent consumers from being misinformed by advertisement and non-scientific sources.

Although CBD-containing foods are currently not legal, a variety of illegally marketed products declared as foods, food supplements, or otherwise intended for oral consumptions can be purchased from several sources – especially online. Beyond potential risks of CBD, the quality of such products may vary. This is of particular note, as almost half of the respondents assume that CBD-containing products have been tested to ensure their safety. However, this is generally not the case for consumer products and foods including food supplements, since these product categories are not tightly regulated by official bodies. In addition to targeted information about potential health risks, clarifying the regulatory circumstances can enable consumers to make an informed decision about whether or not to use CBD-containing products.

### Strengths and limitations

One of the study’s strength is its sample size and accounting for various socio-demographic variables. Using a two-stage study design, we were able to assess the usage rate for CBD-containing products based on a population representative sample (study part I), while focusing on more detailed information on usage and perception for specific groups of users vs. non-users within the study part II. However, since the survey for study part II was conducted via an online questionnaire and using quota sampling, generalizability of our findings is restricted to the characteristics of the recruited sample (e.g., people with low affinity for online applications or without internet access are underrepresented). Also, as participants self-reported their behavior, there might be a bias in their answers due to desirability. As this has been a cross-sectional study, the data represent a selective moment. Statements about trends are therefore not possible.

## Conclusion

About 40% of the German population have already heard of CBD-containing products and about 11% have actually used them. However, as already observed in previous studies, it may be concluded from data in the present study that consumers are insufficiently informed about CBD-containing products. This includes regulatory aspects – but even more important – awareness of potential health risks and the insufficient scientific data basis regarding health benefits. It appears that consumers may underestimate health risks while overestimating beneficial effects. Independent consumer information is highly needed.

### Supplementary Information

**Additional file 1.**

## Data Availability

The datasets generated and analyzed during the current study are not publicly available due to institutional restrictions. Information on data are available from the corresponding author on reasonable request.

## Abbreviations

CBD: Cannabidiol
EU: European Union
M: Means
SD: Standard deviation
THC: Tetrahydrocannabinol

## References

[1] Manthey J (2019). Cannabis use in Europe: current trends and public health concerns. Int J Drug Policy.

[2] Lange BM, Zager JJ (2022). Comprehensive inventory of cannabinoids in Cannabis sativa L.: can we connect genotype and chemotype?. Phytochem Rev.

[3] Zheljazkov VD, Sikora V, Dincheva I (2020). Industrial, CBD, and wild hemp: how different are their essential oil profile and antimicrobial activity?. Molecules.

[4] Huestis MA, Solimini R, Pichini S (2019). Cannabidiol adverse effects and Toxicity. Curr Neuropharmacol.

[5] European Court of Justice. Judgment of the Court (Fourth Chamber) of 19 November 2020 (request for a preliminary ruling from the Cour d’appel d’Aix-en-Provence – France). 2020. Available from: https://curia.europa.eu/juris/document/document.jsf?docid=236909&mode=req&pageIndex=1&dir=&occ=first&part=1&text=&doclang=DE&cid=202842#1. Last Accessed 26 Jan 2023.

[6] de Almeida DL, Devi LA (2020). Diversity of molecular targets and signaling pathways for CBD. Pharmacol Res Perspect.

[7] European Parliament and the Council (2001). Directive 2001/83/EC of the European Parliament and of the Council of 6 November 2001 on the Community code relating to medicinal products for human use. Official J Eur Communities.

[8] European Commission. Novel food catalogue. 2022. Available from: https://webgate.ec.europa.eu/fip/novel_food_catalogue/#. Last Accessed 22 Jul 2022.

[9] European Parliament and the Council (2015). Regulation (EU) 2015/2283 of the European Parliament and of the Council of 25 November 2015 on novel foods, amending Regulation (EU) No 1169/2011 of the European Parliament and of the Council and repealing Regulation (EC) No 258/97 of the European Parliament and of the Council and Commission Regulation (EC) No 1852/2001. OJEU.

[10] European Commission. Summary of applications and notifications. 2022. Available from: https://food.ec.europa.eu/safety/novel-food/authorisations/summary-applications-and-notifications_en . Last Accessed 17 Aug 2022.

[11] European Food Safety Authority (EFSA), Panel on Novel Foods and Food Allergens (NDA) (2022). Statement on safety of cannabidiol as a novel food: data gaps and uncertainties. EFSA J.

[12] Verbraucherzentrale. CBD-Öl legal auf dem Markt? 2022. Available from: https://www.klartext-nahrungsergaenzung.de/wissen/lebensmittel/nahrungsergaenzungsmittel/cbdoel-legal-auf-dem-markt-37660. Accessed 10 Nov 2022.

[13] Kish L (1949). A Procedure for Objective Respondent selection within the Household. J Am Stat Assoc.

[14] Gabler S, Kolb J-P, Sand M, et al. Weighting GESIS Survey Guidelines. 2016. 10.15465/gesis-sg_en_007.

[15] Rao JNK, Scott AJ (1984). On Chi-Squared tests for Multiway Contingency Tables with cell proportions estimated from Survey Data. Ann Stat.

[16] Casanova C, Ramier C, Fortin D (2022). Cannabidiol use and perceptions in France: a national survey. BMC Public Health.

[17] Alayli AFG, Kotz D, Kastaun S (2022). Recreational cannabidiol: awareness, prevalence of use, and Associated Factors in a Representative Sample of the German Population. Subst Use Misuse.

[18] Bhamra SK, Desai A, Imani-Berendjestanki P (2021). The emerging role of cannabidiol (CBD) products; a survey exploring the public’s use and perceptions of CBD. Phytother Res.

[19] Goodman S, Wadsworth E, Schauer G (2022). Use and perceptions of Cannabidiol Products in Canada and in the United States. Cannabis Cannabinoid Res.

[20] US Food and Drug Administration (FDA). FDA regulation of cannabis and cannabis-derived products, including cannabidiol (CBD). 2021. Available from: https://www.fda.gov/news-events/public-health-focus/fda-regulation-cannabis-and-cannabis-derived-products-including-cannabidiol-cbd#othercbdapproved. Last Accessed 21 Jul 2022.

[21] Health Canada. Final regulations: edible cannabis, cannabis extracts, cannabis topicals. 2019. Available from: https://www.canada.ca/content/dam/hc-sc/documents/services/drugs-medication/cannabis/resources/final-regulations-edible-cannabis-extracts-topical-eng.pdf. Accessed 22 July 2022.

[22] Wheeler M, Merten JW, Gordon BT (2020). CBD (Cannabidiol) product attitudes, knowledge, and use among young adults. Subst Use Misuse.

[23] German Federal Institute for Risk Assessment. Cases of Poisoning: liquids containing cannabidiols for e-cigarettes can be manipulated. Opinion of the BfR 2021;no 005/2020 issued 23 January 2020, 10.17590/20200206-105237.

[24] Li J, Carvajal R, Bruner L (2021). The current understanding of the benefits, safety, and regulation of cannabidiol in consumer products. Food Chem Toxicol.

[25] European Medicines Agency (EMA). Epidyolex (cannabidiol) - An overview of Epidyolex and why it is authorised in the EU. 2021. Available from: https://www.ema.europa.eu/en/documents/overview/epidyolex-epar-medicine-overview_en.pdf. Accessed 19 Aug 2022.

[26] Peng J, Fan M, An C (2022). A narrative review of molecular mechanism and therapeutic effect of cannabidiol (CBD). Basic Clin Pharmacol Toxicol.

[27] Khalsa JH, Bunt G, Blum K (2022). Review: cannabinoids as medicinals. Curr Addict Rep.

