# Supplementary material for: Usage and health perception of cannabidiol-containing products among the population in Germany: a descriptive study conducted in 2020 and 2021
Source: BMC Public Health. 2023 Nov 23;23:2318. doi: 10.1186/s12889-023-17142-0 (PMC10666397; doi:10.1186/s12889-023-17142-0)
Supplement: Supplementary file 1 — Additional file 1. [file 12889_2023_17142_MOESM1_ESM.pdf]

Supplement A: Questionnaires

Note: In both study parts, a questionnaire in German language was used. The English translation of the questions is provided below.

Q1: Questionnaire study part I

Question – Awareness

Now we would like to ask you a question about cannabidiol. Cannabidiol is also known as CBD.

Have you already heard of products containing CBD?

1 Yes, I have already heard of it
2 No, I have not heard of it

99 don't know

Question – CBD use

Have you already consumed or used products containing CBD?

1 Yes, I have already consumed or used it.
2 No, I have not consumed or used it yet

99 don't know

Q2: Questionnaire study part II

Question – Awareness

Have you heard of the following products?
(Items are randomised)

(1) Products containing CBD (cannabidiol)
(2) Products containing THC (tetrahydrocannabidiol)
(3) Products containing BPS (bisphenol A)
(4) Products containing aluminium salts
(5) Products containing Arkonital

1 Yes, I have heard of it
2 No, I have not heard of it

99 don't know / no information

Question – CBD use

Have you already consumed or used products containing CBD (cannabidiol)?

1 Yes, I have already consumed/used it.
2 No, I have not consumed/used yet
3 I am not sure

99 No answer

Question – Age
How old are you?
Open question

Question – Gender

Your gender - you are:

1 male
2 female
3 diverse

Question – School-leaving qualification

What is your highest educational qualification?
If you are still in school, please indicate which degree you are aiming for.

1 Without school leaving certificate
2 Low secondary education
3 Mediate secondary education
4 High secondary education
5 Tertiary education

Question – Product groups

To which product groups can you assign the products that you have already consumed or used?
Note: Please select all that apply.

(Items are randomised, except 98 and 99)

1 CBD oil or tinctures (taken orally)
2 CBD crystals
3 CBD capsules or pills
4 CBD chewing gum or dragées
5 CBD patches
6 CBD flowers
7 CBD-containing foods (e.g. chocolate, cereal bars)
8 Cosmetics or care products containing CBD
9 CBD-containing liquids for e-cigarettes or vaporisers
10 Beverages containing CBD
11 CBD-containing medicines/drugs

98 Other, namely __________
99 don't know / no information

Question – Reason for use

For what reasons have you consumed or used products containing CBD (cannabidiol)?
Note: Please write the answer in the text box.

____

99 no answer

Question – Frequency of use

How often do you currently consume or use products containing CBD (cannabidiol)?

1 daily
2 several times a week
3 about once a week
4 about once or three times a month
5 less than once a month
6 not at all

99 don't know / no answer

Question – Intention of use

*C*an you imagine consuming or using products containing CBD (cannabidiol) in the future?

1 Yes
2 No

99 don't know / no answer

Question – Acquisition

Where do you generally purchase products containing CBD (cannabidiol)?
Note: Multiple answers allowed.

(Items are randomised, except 7, 97 and 99)

1 Online shop
2 Drugstore
3 Pharmacy
4 Supermarket/discounter
5 Organic market/reform store
6 Specialised shop for CDB or hemp products
7 Other shop

97 Other way of purchase, namely: _________
99 don't know / no answer

Question – Assessment of risk-benefit

How do you assess the health risk and health benefit of products containing CBD (cannabidiol)?

(Items are randomised)

(1) Health risk
(2) Health benefit

1 1 - very low
2 2 - low
3 3 - neither
4 4 - high
5 5 - very high

99 don't know / no answer

Question – Health risk mentioned

Which health risks do you see in products containing CBD (cannabidiol)?
Note: Please write the answer in the text box.

____

99 no answer

Question – Health benefits mentioned

Which health benefits do you see in products containing CBD (cannabidiol)?
Note: Please write the answer in the text box.

____

99 no answer

Question – Knowledge

In your opinion, does each of the following statements apply or not?

(Items are randomised)

(1) Products containing CBD (cannabidiol) have been tested for health safety.
(2) The substance CBD (cannabidiol) can be physically addictive.
(3) The substance CBD (cannabidiol) can have an intoxicating effect ("high").
(4) Products containing CBD (cannabidiol) can influence the effect of medication.
(5) Products containing CBD (cannabidiol) may also contain THC.

1 Does not apply
2 Does apply

99 don't know / no answer

SUPPLEMENTAL B: Tables

Table S1. Interpretation of used effect sizes

	Small effect	Medium effect	Large effect
Cohen's *d*	|0.20|	|0.50|	|0.80|
Cramer's *V* (*df* = 1)	0.10	0.30	0.50
Cramer's *V* (*df* = 2)	0.07	0.21	0.35

Note. Rules of thumb for interpretation of effect sizes based on Cohen (1988).[1]

Table S2. Demographic characteristics of respondents in study part I

	Total		Gender				Highest educational level						Age (in years)					
			Female		Male		Student / Lower secondary school		Secondary school without high school diploma		High school / University degree		14 to 29		30 to 59		60 and older	
	n	%	*n*	%	*n*	%	*n*	%	*n*	%	*n*	%	*n*	%	*n*	%	*n*	%
Base	1,011	100	510	50.5	501	49.5	378	37.4	306	30.2	327	32.4	198	19.6	483	47.8	330	32.6
Have heard of CBD[a]	406	40.2	203	50.1	203	49.9	109	26.8	132	32.5	165	40.7	123	30.2	206	50.7	78	19.1
Have used/ consumed CBD[b]	116	11.4	51	43.8	65	56.2	31	26.6	31	26.5	54	46.9	34	29.6	65	56.5	16	13.9

[a] Reflects the number and percentage of participants answering "yes" to the question (1) "Have you already heard of any products that contain CBD?"
[b] Reflects the number and percentage of all participants answering "yes" to the question (1) and subsequently to question (2) "Have you already consumed or used any products that contain CBD?"

Table S3. Demographic characteristics of respondents in study part II

	Total		Gender						Highest educational level						Age (in years)					
			Female		Male		Divers		Without graduation / Lower secondary school		Secondary school without high school diploma		High school / university degree		16 to 29		30 to 59		60 and older	
	n	%	*n*	%	*n*	%	*n*	%	*n*	%	*n*	%	*n*	%	*n*	%	*n*	%	*n*	%
Base	2,000	100.0	1,057	52.9	941	47.1	2	0.1	421	21.2	712	35.6	867	43.4	562	28.1	1,027	51.4	411	20.5
Have used/consumed CBD[a]	535	26.8	277	51.8	256	47.9	2	0.4	113	21.1	182	34.0	240	44.9	196	36.6	254	47.5	85	15.9
Have not used/consumed CBD[b]	1,271	63.6	695	54.7	576	45.3	0	0.0	260	20.5	463	36.4	548	43.1	298	23.4	690	54.3	283	22.3
Not sure[c]	186	9.3	84	45.2	102	54.8	0	0.0	45	24.2	63	33.9	78	41.9	64	34.4	80	43.0	42	22.6
No response[d]	8	0.4	1	12.5	7	87.5	0	0.0	3	37.5	4	50.0	1	12.5	4	50.0	3	37.5	1	12.5

[a] Reflects the number and percentage of all participants answering "yes" to the question "Have you already consumed or used any products that contain CBD?"
[b] Reflects the number and percentage of all participants answering "no" to the question "Have you already consumed or used any products that contain CBD?"
[c] Reflects the number and percentage of all participants answering "I am not sure" to the question "Have you already consumed or used any products that contain CBD?"
[d] Reflects the number and percentage of all participants answering "no response" to the question "Have you already consumed or used any products that contain CBD?"

Table S4. Intention of future use (study part II)

Can you imagine consuming or using products that contain CBD (cannabidiol) in the future?	*n*	%
Yes	614	48.3
No	324	25.5
Don't know / no response	333	26.2
**Total**	**1271**	**100.0**

Base: Respondents who have not consumed/used CBD (n = 1,271).

Table S5. Source of purchase (study part II)

Where do you generally purchase products containing CBD (cannabidiol)? (multiple response)	*n*	%
Online shop	296	55.3
Pharmacy	113	21.1
Specialized shops for CBD- and hemp-products	107	20.0
Drug store	105	19.6
Supermarket / discount store	62	11.6
Organic market / health food store	54	10.1
Other shop	31	5.8
Others	21	3.9
Don't know / no response	25	4.7
**Total**	**535**	**100**

Base: Respondents who have already consumed/used CBD (n = 535).

Table S6. Frequency of use by age and education (study part II)

How often do you currently consume or use products containing CBD (cannabidiol)?	Total		Age (in years)						Highest educational level					
			16 to 29		30 to 59		60 and older		Without graduation/ Lower secondary school		Secondary school without high school diploma		High school/university degree	
	n	%	*n*	%	*n*	%	*n*	%	*n*	%	*n*	%	*n*	%
daily	94	17.6	14	7.1	55	21.7	25	29.4	33	29.2	34	18.7	27	11.3
several times a week	88	16.4	24	12.2	43	16.9	21	24.7	29	25.7	26	14.3	33	13.8
about once a week	43	8.0	20	10.2	17	6.7	6	7.1	4	3.5	17	9.3	22	9.2
about once or three times a month	75	14.0	32	16.3	35	13.8	8	9.4	7	6.2	27	14.8	41	17.1
less than once a month	125	23.4	65	33.2	51	20.1	9	10.6	20	17.7	38	20.9	67	27.9
not at all	106	19.8	37	18.9	53	20.9	16	18.8	20	17.7	39	21.4	47	19.6
don’t know / no response	4	0.7	4	2.0	0	0.0	0	0.0	0	0.0	1	0.5	3	1.3
**Total**	**535**	**100.0**	**196**	**100.0**	**254**	**100.0**	**85**	**100.0**	**113**	**100.0**	**182**	**100.0**	**240**	**100.0**

Base: Respondents who have already consumed/used CBD (n = 535).

Table S7. Perceived health risks and health benefits by usage of CBD-containing products (study part II)

	Health risks						Health benefits					
	Total		Users		Non-users		Total		Users		Non-users	
	n	%	*n*	%	*n*	%	*n*	%	*n*	%	*n*	%
Very low	467	23.4	223	41.7	204	16.1	66	3.3	26	4.9	37	2.9
Low	626	31.3	168	31.4	397	31.2	180	9.0	39	7.3	126	9.9
Neither	423	21.2	102	19.1	280	22.0	391	19.6	100	18.7	250	19.7
High	151	7.6	13	2.4	120	9.4	754	37.7	205	38.3	479	37.7
Very high	65	3.3	11	2.1	49	3.9	352	17.6	156	29.2	161	12.7
Don't know / no response	268	13.4	18	3.4	221	17.4	257	12.9	9	1.7	218	17.2
**Total**	**2,000**	**100.0**	**535**	**100.0**	**1,271**	**100.0**	**2,000**	**100.0**	**535**	**100.0**	**1,271**	**100.0**

References

1. Cohen, J. (1988). Statistical Power Analysis for the Behavioral Sciences (2nd ed.). New York: Routledge Academic. https://doi.org/10.4324/9780203771587
